# Hydrogen-Rich Saline Attenuates Cardiac and Hepatic Injury in Doxorubicin Rat Model by Inhibiting Inflammation and Apoptosis

**DOI:** 10.1155/2016/1320365

**Published:** 2016-12-26

**Authors:** Yunan Gao, Hongxiao Yang, Yanbin Fan, Lin Li, Jiahui Fang, Wei Yang

**Affiliations:** ^1^Department of Cardiology, The Fourth Affiliated Hospital of Harbin Medical University, 37 Yiyuan Street, Harbin, Heilongjiang 150001, China; ^2^Department of Cardiology, The First Affiliated Hospital of Harbin Medical University, 23 Youzheng Street, Harbin, Heilongjiang 150001, China

## Abstract

Doxorubicin (DOX) remains the most effective anticancer agent which is widely used in several adult and pediatric cancers, but its application is limited for its cardiotoxicity and hepatotoxicity. Hydrogen, as a selective antioxidant, is a promising potential therapeutic option for many diseases. In this study, we found that intraperitoneal injection of hydrogen-rich saline (H_2_ saline) ameliorated the mortality, cardiac dysfunction, and histopathological changes caused by DOX in rats. Meanwhile, serum brain natriuretic peptide (BNP), aspartate transaminase (AST), alanine transaminase (ALT), albumin (ALB), tissue reactive oxygen species (ROS), and malondialdehyde (MDA) levels were also attenuated after H_2_ saline treatment. What is more, we further demonstrated that H_2_ saline treatment could inhibit cardiac and hepatic inflammation and apoptosis relative proteins expressions by western blotting test. In conclusion, our results revealed a protective effect of H_2_ saline on DOX-induced cardiotoxicity and hepatotoxicity in rats by inhibiting inflammation and apoptosis.

## 1. Introduction

Anthracyclines remain the most widely prescribed and effective anticancer agents. Doxorubicin (DOX), an anthracycline anticancer drug of secondary metabolite of* Streptomyces peucetius* var.* caesius*, is widely used in several adult and pediatric cancers such as thyroid cancer, ovarian cancer, leukemia, lymphomas, and breast cancer [1]. But the application is limited for its cytotoxicity in normal organs like heart and liver [2, 3].

Anthracycline cardiotoxicity is exponentially dose-dependent, with an average incidence of 5.1% at 400 mg/m^2^ that becomes higher above 500 mg/m^2^, albeit with substantial individual variation. Cardiomyopathy may develop at lower doses in the presence of risk factors like age, hypertension, arrhythmias, coronary disease, and so forth [4, 5]. Using clinical criteria, adult survivors of childhood cancer with a median time from diagnosis of 25 years (range: 10–47 years) were assessed for the prevalence of adverse health outcomes. Among them, cardiac dysfunction reaches up to 56.4% [6, 7]. Doxorubicin-induced cardiomyopathy is a lethal disease, because it may not be detected for many years and remains a lifelong threat. When congestive heart failure develops, mortality is approximately 50% [8].

It has been reported that about 40% of patients suffered liver injury after doxorubicin treatment [9]. It is still a challenge to find a favorable treatment for prevention of the toxicity or intervention after toxicity develops [10].

Now it is known that the mechanisms of doxorubicin-mediated cell death include oxidative stress, apoptosis, intracellular calcium dysregulation, topoisomerase II poisoning, DNA adduct formation, and ceramide overproduction [11, 12]. However, it seems that some drugs can protect the organs from the attack of oxidative stress theoretically. Besides, several pharmacologic agents, like antioxidants, hematopoietic cytokines, and iron-chelating agents, are reported to be used to reduce the toxic effects to some extent [13, 14]. However, the exact mechanism of cardiotoxicity and hepatotoxicity induced by doxorubicin remains poorly understood.

Hydrogen (H_2_), the most abundant and smallest element in the universe, has advantageous distribution characteristics for its capability to penetrate biomembranes and diffuse into the organelles and nucleus [15]. In 2007, Ohsawa et al. [16] used an acute rat model in which oxidative stress damage was induced in the brain by focal ischemia and reperfusion; they found that hydrogen acts as a therapeutic antioxidant by selectively reducing cytotoxic oxygen radicals, especially hydroxyl radical (OH^•^), the most cytotoxic reactive oxygen species (ROS). Overproduction of reactive oxygen can result in not only direct organ injury but also exacerbation of the inflammatory reaction simultaneously. The release of proinflammatory cytokines and chemokines, including tumor necrosis factor-*α* (TNF-*α*), interleukin-1*β* (IL-1*β*), and interleukin-6 (IL-6), the most important cytokines mediating the inflammatory response, normally triggers beneficial host innate immune response to confine tissue damage [17]. Nowadays, the inhibition of inflammation by hydrogen makes sense. Its rapid gaseous diffusion makes it highly effective for reducing cytotoxic radicals, and it has been proven to be protective against injury to various organs including the brain, liver, heart, and lung [18–21].

Studies have confirmed that DOX-derived ROS could act as an intrinsic stress that activates mitogen activated protein kinases (MAPK), p38, JNK, and NF-*κ*B pathways as well as intracellular p53 accumulation, leading to an increase in proinflammatory cytokines (TNF-*α* and IL-1*β*) and alteration in the ratio of proapoptotic proteins to antiapoptotic proteins (e.g., Bax to Bcl-2), cytochrome C (Cyto C) release, and caspase-3 (C3) activation [22–24]. The present study aims to investigate the potential anti-inflammation and antiapoptosis effect of hydrogen-rich saline on DOX-induced cardiotoxicity and hepatotoxicity in Wistar albino rats.

## 2. Materials and Methods

### 2.1. Animals

Ninety male Wistar albino rats (Changsheng Biotechnology Co. Ltd., Liaoning, China) weighing an average of 200 g were used in this study in accordance with the Guidelines of Laboratory Animals of the First Affiliated Hospital of Harbin Medical University's protocol for care and use. They were housed with free access to food and water in a rodent facility under 12 h light-dark cycle and the temperature of 20–25°C. All rats were acclimated for seven days prior to any experimental procedures.

### 2.2. Preparation and Estimation of Hydrogen-Rich Saline

Hydrogen, produced from a hydrogen generator (HA300, Dura Safer Technology, Ltd., Beijing, China), was dissolved in normal saline in a thick plastic bag with no dead volume until reaching 4 atm for 1 hour. It was prepared freshly and sterilized by gamma radiation before injection. The concentration of hydrogen-rich saline was measured by oxidimetry method with a reagent containing methylene blue and colloidal platinum (Miz Company, Kanagawa, Japan) [25], and it was maintained at about 0.55 mmol/L to keep the concentrations of hydrogen in the heart and liver above 20 ppb/g [26].

### 2.3. Experimental Protocol

Ninety rats were divided into three groups at random as the normal-saline group (NS, *n* = 30), the doxorubicin group (DOX, *n* = 30), and the doxorubicin plus hydrogen-rich saline group (HS, *n* = 30). The DOX group and the HS group were treated by intraperitoneal injection with a dose of 2 mg/kg doxorubicin (Adriamycin®, Pfizer, Nerviano (MI), Italy) every 3 days for 30 days while the NS group was given saline of the same dose by the same way. Meanwhile, the HS group was given intraperitoneal injection of hydrogen-rich saline (10 mL/kg) but the other two groups were given the same dose of normal saline every day. On the 30th day, all rats were sacrificed by euthanasia to collect the blood and tissue samples.

### 2.4. Echocardiography

On the 30th day, the rats were subjected to induction of anesthesia at a concentration of 4% and maintained deeply anesthetized at a concentration of 2% with isoflurane (R510-22, RWD Life Science, Co. Ltd., Shenzhen, China). Transthoracic echocardiography was performed by an experienced ultrasonographic doctor who was blinded to the grouping of the rats. The interventricular septal thickness at diastole (IVSd), left ventricular internal diameter in diastole and systole (LVDd and LVDs), left ventricular posterior wall at diastole (LVPWd), ejection fraction (EF), and shortening fraction (FS) of each rat were assessed using a 12 MHz transducer connected to a commercially available echocardiographic system (SONOS 7500, Philips). All measurements are averages from three consecutive cardiac cycles.

### 2.5. Serum Parameter

Blood samples of all survived rats from aorta were collected into heparin-containing tubes, centrifuged at 3000*g* for 15 min at 4°C, and measured within 2 hours. The serum concentrations of BNP were detected using ELISA kits in accordance with the manufacturers' instructions (Nanjing Jiancheng Bioengineering Institute, Nanjing, China). Serum concentrations of AST, ALT, and ALB in different treatment groups were measured by an automatic biochemical analyzer (TMS-1024, Tokyo, Japan).

### 2.6. ROS and MDA Levels of Tissue

After euthanasia, cardiac and hepatic tissues of all survived rats were, respectively, washed in icy phosphate buffer saline. ROS was quantified by ELISA (Lanpai Biotech. Co. Ltd., Shanghai, China). MDA concentration, a presumptive marker of oxidant-mediated lipid peroxidation, was measured using a commercial kit (KeyGEN Biotech. Co. Ltd., Nanjing, China).

### 2.7. Histological Study

After blood sample collection, the rats were sacrificed and their hearts and livers were rapidly excised for histopathological and biochemical analyses. Tissues were fixed with 10% buffered formalin, embedded in paraffin, sectioned into 2 *μ*m thick sections, and stained with hematoxylin and eosin (H&E). 10 random fields at 400x magnification in each specimen were observed and photographed with a light microscope (DP73, Olympus Co., Japan) by 3 pathologists of blinded method.

### 2.8. Terminal Deoxynucleotidyl Transferase-Mediated dUTP-Biotin End Labeling (TUNEL) Staining Method

The TUNEL assay was performed to label the 3′-end of fragmented DNA in tissue sections according to the manufacturer's instructions (Roche, Switzerland) and stained with DAB kit (ZSGB-BIO, China). Stained cells area was counted on 10 random sections of liver from each rat without knowledge of the group of rats from which the tissue was taken. The TUNEL-positive area was expressed as a percentage of the total area. Finally, slides were examined by a light microscope (DP73, Olympus Co., Japan), and quantitative statistical analysis was performed with the KS400 Image Analysis System (KS400, Zeiss, Germany).

### 2.9. Assessment of Inflammation and Apoptosis

Western blotting was performed according to the commercial instruction. Total proteins were extracted from the tissues with the lysis buffer for protein immunoblotting. Protein concentrations were measured by BCA protein assay kit with bovine serum albumin (BSA) as standard (Beyotime, China). Protein samples were separated in each well of 12.5% sodium dodecylsulfate-polyacrylamide gel electrophoresis (SDS-PAGE) and blotted to Polyvinylidene fluoride (PVDF) membranes. The blots were blocked with 5% fat-free milk for 1 h at room temperature and then probed with primary antibodies including TNF-*α* (1 : 500 dilution, number ab6671, Abcam), IL-1*β* (1 : 1000 dilution, number ab9722, Abcam), IL-6 (1 : 500 dilution, number MAB5011, R&D), Bax (1 : 1000 dilution, number ab182733, Abcam), Bcl-2 (1 : 1000 dilution, number ab59348, Abcam), cleaved caspase-8 (C8) (1 : 1000 dilution, NB100-56116SS, NOVUS), cleaved caspase-3 (C3) (1 : 1000 dilution, number Asp175, CST), and *β*-actin (1 : 1000 dilution, number TA-09, ZSGB). They were incubated at 4°C overnight. The membranes were washed with TBS-T and then incubated with horseradish peroxidase-conjugated secondary antibody (1 : 2000 dilution; ZB-2301, ZB-2305, and ZSGB) for 1 hour at room temperature. Finally, the bands were collected by Imaging System (Bio-Rad, Hercules, CA, USA). *β*-Actin was used as the control for equal loading of the protein.

### 2.10. Data Processing and Statistical Analysis

Recipient survival was plotted using the Kaplan–Meier method and was analyzed using the log-rank test. Quantitative data were expressed as mean ± standard deviation (SD). Analysis of variance (one-way ANOVA) was used for multiple comparisons, with a posttest of Student-Newman-Keuls. Statistical significance was considered at a *P* value of <0.05. Statistical analyses were performed using SPSS software (SPSS Inc., Chicago, USA).

## 3. Results

### 3.1. Effects of Hydrogen-Saline Treatment on Mortality, Cardiac Dysfunction, and Pathological Changes

**Figure 1:**
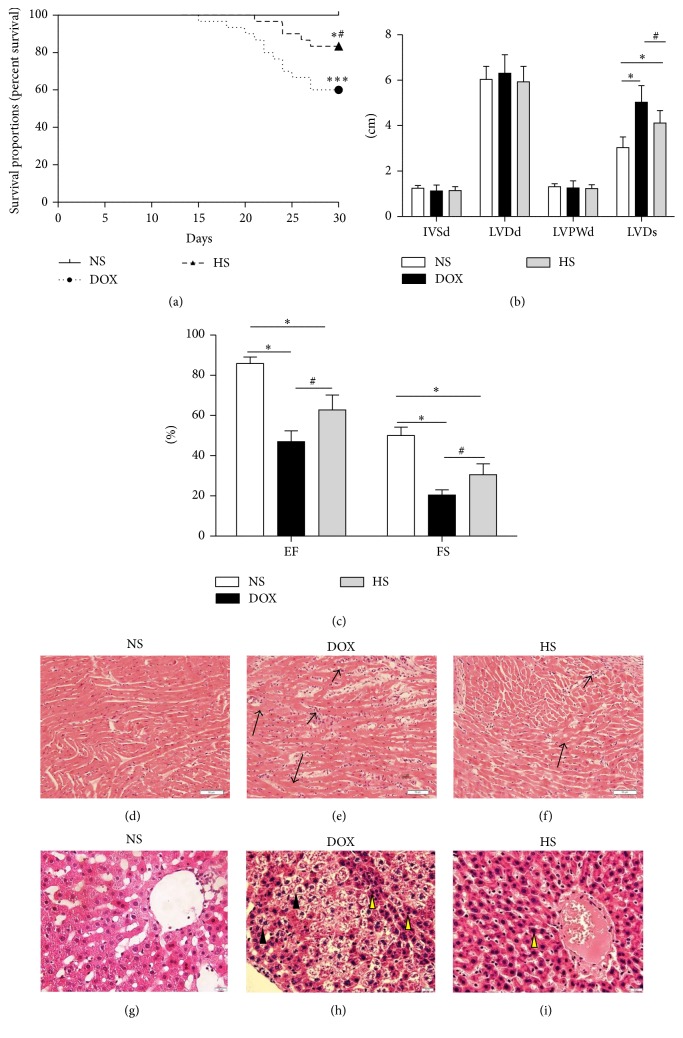
Effects of hydrogen-rich saline treatment on mortality, cardiac dysfunction, and pathological changes. Kaplan–Meier analyses of cumulative survival at 30 days after different treatments (a). The IVSd, LVDd, LVPWd, LVDs, EF, and FS of each rat were assessed ((b) and (c)). Morphologic changes of the heart (200x magnification; (d–f)) and liver (400x magnification; (g–i)) were processed for HE staining at 30 days (short arrows for infiltrated inflammatory cells and long arrows for focal myolysis; yellow arrowheads for karyopyknosis and black arrowheads for vacuolar degeneration). ^*∗*^ *P* < 0.05 versus NS group; ^*∗∗∗*^ *P* < 0.001 versus NS group; ^#^ *P* < 0.05 versus DOX group.

By the end of the 30th day, all 30 rats in the NS group were alive, while 18 out of 30 (60%) rats in the DOX group (*P* < 0.05 versus NS group; Figure 1(a)) and 25 out of 30 (83.33%) rats survived in the HS group (*P* < 0.05 versus DOX group; Figure 1(a)). The LVD was significantly increased in the DOX group (*P* < 0.05; Figure 1(b)), whereas it was dramatically reduced by the hydrogen-rich saline treatment (*P* < 0.05; Figure 1(b)). However, there were no noted differences between the three groups about the IVSd, LVDd, and LVPWd (*P* > 0.05; Figure 1(b)). In addition, the EF and FS of DOX group were notably decreased compared with the NS group (*P* < 0.05; Figure 1(c)), but they were both remarkably increased in the HS group (*P* < 0.05; Figure 1(c)). Representative histological sections are shown in Figures 1(d)–1(f) (heart) and Figures 1(g)–1(i) (liver). Histopathological results from the H&E light micrographs showed that the NS group showed normal cardiac and hepatic architecture, and pathological injuries were obviously found in the DOX group, including infiltration of inflammatory cells, focal myolysis, karyopyknosis, and vacuolar degeneration, while they were significantly ameliorated in the HS group. According to these results, we further investigated the protective effect of H_2_ saline on serum parameters of heart and liver injuries.

### 3.2. Effect of Hydrogen-Saline on Serum Parameters

**Figure 2:**
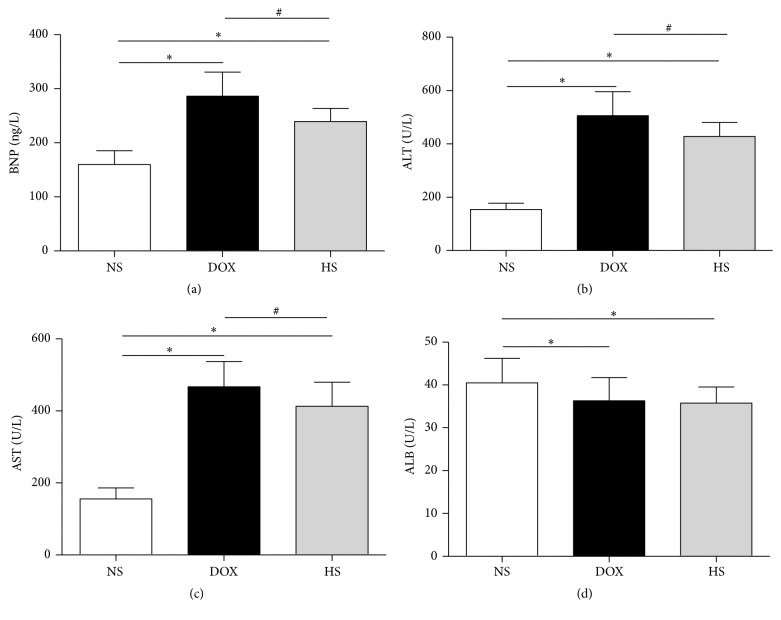
Effect of hydrogen-rich saline on serum parameters. Serum BNP (a), ALT (b), AST (c), and ALB (d) levels in three groups were detected. Data are shown as mean ± SD. ^*∗*^ *P* < 0.05 versus NS group; ^#^ *P* < 0.05 versus DOX group.

Compared with NS group, serum BNP, ALT, and AST levels of DOX group were significantly increased (*P* < 0.05; Figures 2(a)–2(c)), but they were all remarkably reduced in the HS group (*P* < 0.05; Figures 2(a)–2(c)). Although DOX treatment decreased serum ALB levels (*P* < 0.05; Figure 2(d)), H_2_ saline treatment was of no significance compared with the DOX group (*P* > 0.05; Figure 2(d)). These data suggest that hydrogen-rich saline effectively protects heart and liver function against doxorubicin-induced cardiotoxicity and hepatotoxicity. In addition, we investigated the ROS and MDA levels of cardiac and hepatic tissues because of the proven antioxidation of hydrogen.

### 3.3. Effect of Hydrogen-Saline Treatment on ROS and MDA Levels

**Figure 3:**
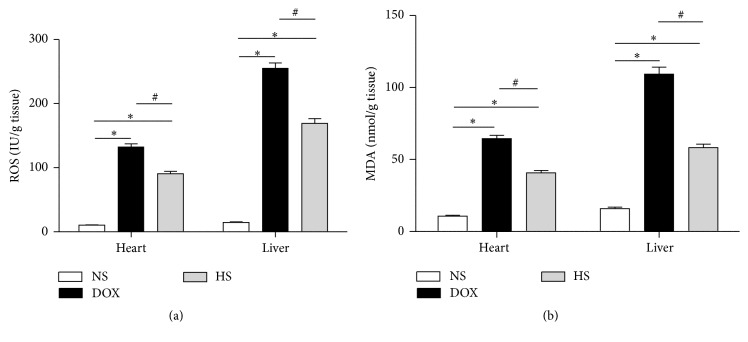
Effects of hydrogen-rich saline on ROS and MDA levels. The ROS (a) and MDA (b) levels of cardiac and hepatic tissues in three groups were detected. Data are shown as mean ± SD. ^*∗*^ *P* < 0.05 versus NS group; ^#^ *P* < 0.05 versus DOX group.

ROS and MDA levels in cardiac and hepatic tissue were measured, showing that both ROS and MDA levels were significantly higher in the DOX group when compared with the NS group (*P* < 0.05; Figures 3(a) and 3(b)). In addition, they were significantly reduced in the HS group (*P* < 0.05; Figures 3(a) and 3(b)). These findings indicate that hydrogen-rich saline may act as an antioxidant to decrease the cardiac and hepatic ROS and MDA levels. Because oxidative stress injury could induce inflammation and apoptosis, we further investigated the changes of inflammation and apoptosis protein levels by western blotting.

### 3.4. The Anti-Inflammatory Effect of Hydrogen-Saline Treatment on Cardiac and Hepatic Tissue

**Figure 4:**
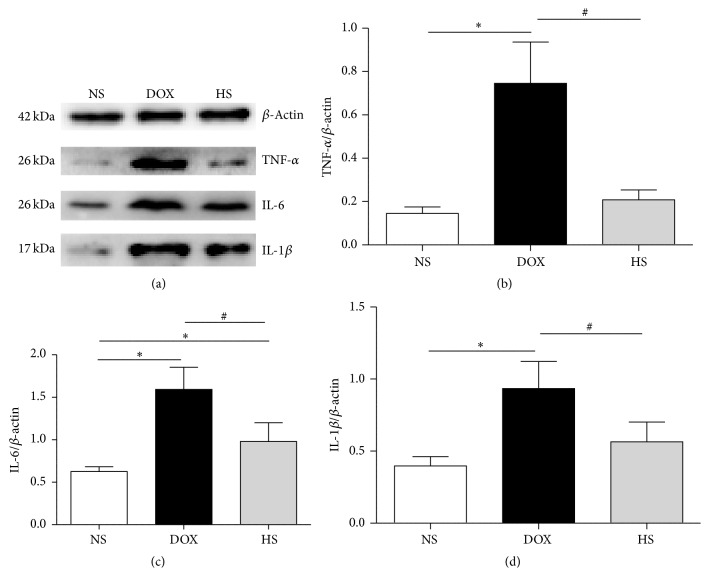
The anti-inflammatory effect of hydrogen-rich saline on cardiac tissue. Representative expression of inflammatory-related proteins in the heart after the treatment was detected (a–d). Data are shown as mean ± SD, *n* ≥ 3. ^*∗*^ *P* < 0.05 versus NS group; ^#^ *P* < 0.05 versus DOX group.

**Figure 5:**
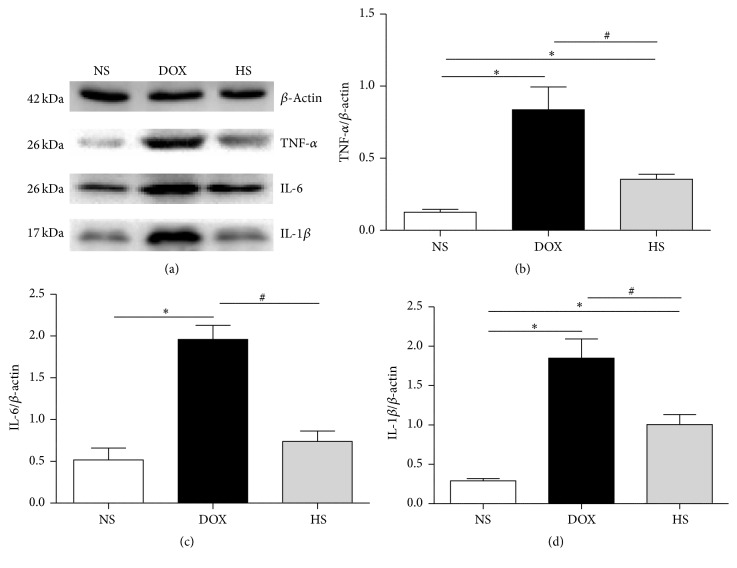
The anti-inflammatory effect of hydrogen-rich saline on hepatic tissue. Representative expression of inflammatory-related proteins in the liver after the treatment was detected (a–d). Data are shown as mean ± SD, *n* ≥ 3. ^*∗*^ *P* < 0.05 versus NS group; ^#^ *P* < 0.05 versus DOX group.

The results of expressions of inflammation relative proteins are presented in Figures 4(a)–4(d) (heart) and Figures 5(a)–5(d) (liver). The expressions of TNF-a, IL-1*β*, and IL-6 in both cardiac and hepatic tissue were markedly increased after doxorubicin injection, and hydrogen-rich saline treatment could reduce the elevation of these inflammatory-related proteins expressions (*P* < 0.05; Figures 4 and 5). These results demonstrate that hydrogen-rich saline protects against heart and liver injury by inhibiting inflammatory responses.

### 3.5. The Antiapoptosis of Hydrogen-Saline Treatment on Cardiac and Hepatic Tissue in TUNEL Staining

**Figure 6:**
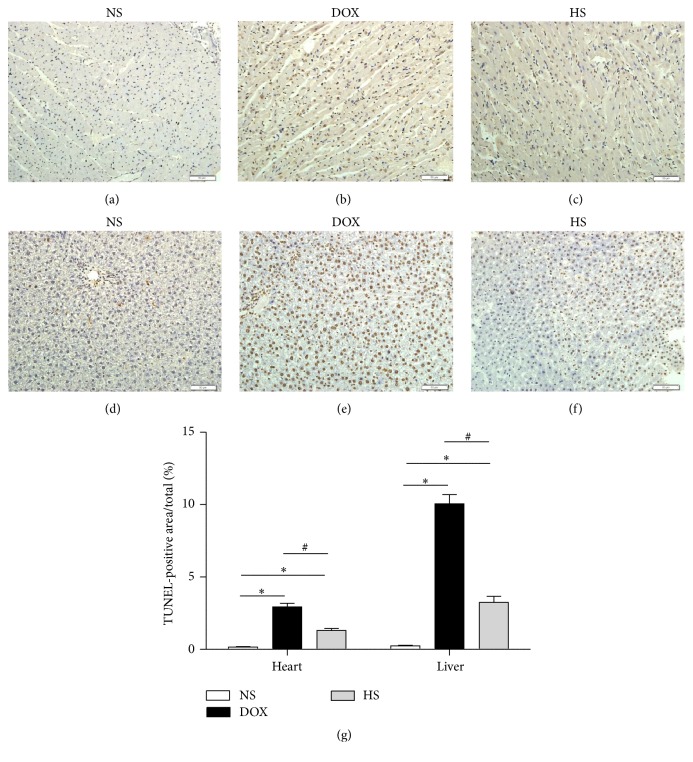
The antiapoptosis of hydrogen-rich saline on cardiac and hepatic tissue in TUNEL staining. Heart (200x magnification; (a–c)) and liver (200x magnification; (d–f)) sections of different treatment groups were stained by TUNEL, and the percentage of TUNEL-positive area (brown staining) of cardiac and hepatic (g) tissues was calculated for each group. Data are shown as mean ± SD. ^*∗*^ *P* < 0.05 versus NS group; ^#^ *P* < 0.05 versus DOX group.

The TUNEL assay findings are shown in Figures 6(a)–6(c) (heart) and Figures 6(d)–6(f) (liver). The percentages of TUNEL-positive area in the cardiac and hepatic (Figure 6(g)) slides were significantly increased in the DOX group (*P* < 0.05), whereas they were obviously reduced in the HS group (*P* < 0.05). These findings demonstrate that hydrogen-rich saline could decrease DOX-induced cell apoptosis.

### 3.6. The Antiapoptosis of Hydrogen-Saline Treatment on Cardiac and Hepatic Tissue

**Figure 7:**
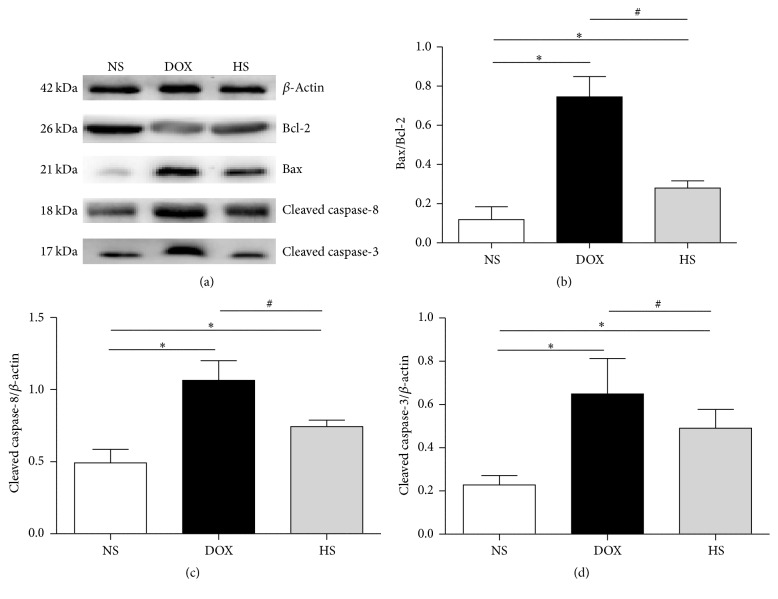
The antiapoptosis effect of hydrogen-rich saline on cardiac tissue. Representative expression of apoptosis-related proteins in the heart of three groups was detected (a–d). Data are shown as mean ± SD, *n* ≥ 3. ^*∗*^ *P* < 0.05 versus NS group; ^#^ *P* < 0.05 versus DOX group.

**Figure 8:**
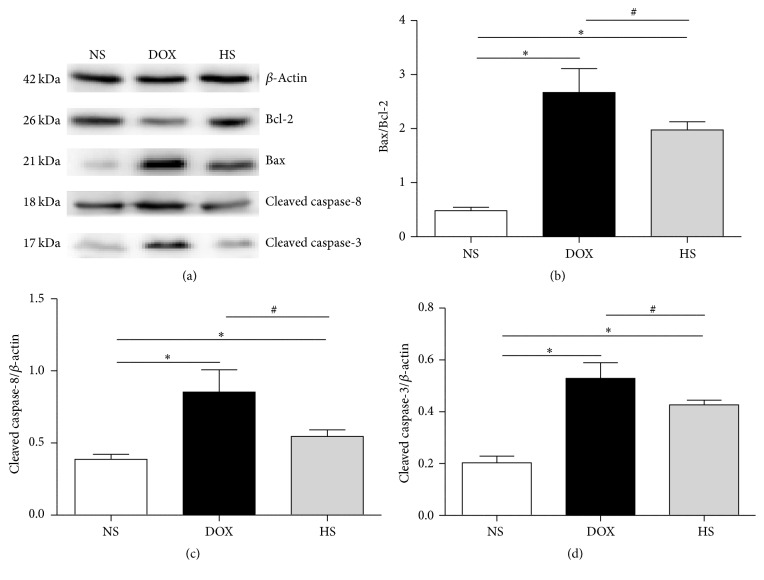
The antiapoptosis effect of hydrogen-rich saline on hepatic tissue. Representative expression of apoptosis-related proteins in the liver of three groups was detected (a–d). Data are shown as mean ± SD, *n* ≥ 3. ^*∗*^ *P* < 0.05 versus NS group; ^#^ *P* < 0.05 versus DOX group.

The results of expressions of apoptosis relative proteins are presented in Figures 7(a)–7(d) (heart) and Figures 8(a)–8(d) (liver). We found that the Bax/Bcl-2, cleaved C8, and cleaved C3 levels were higher in the DOX group compared with the NS group but were significantly reduced in the HS group (*P* < 0.05; Figures 7 and 8). These findings indicate that hydrogen-rich saline protects heart and liver from injury by inhibiting cell apoptosis.

## 4. Discussion

This study demonstrated a protective effect of hydrogen-rich saline on doxorubicin-induced cytotoxicity and hepatotoxicity, such as reduction in mortality, attenuation of heart and liver dysfunction, structural damage, and infiltration of inflammatory cells.

It is known that the main side effect of doxorubicin is the formation of free radicals in normal cells. Considerable evidence has demonstrated the antineoplastic activity by intercalation into DNA structure and production of ROS [27]. Recent studies have reported that hydrogen-rich saline prevents organ injury by decreasing ROS generation [16, 28], same as we detected. What is more, in this study, MDA, which is the end product of oxidative injury and an indicator of lipid peroxidation, rapidly increased both in the cardiac and in the hepatic tissues in the group with doxorubicin treatment. The ROS and MDA levels were both decreased remarkably after hydrogen-rich saline treatment, which indicated that hydrogen-rich saline could reduce oxidative stress induced by doxorubicin. Because hydrogen-rich saline reduced the product of lipid peroxidation, the instability induced by doxorubicin of cellular structures might be ameliorated.

Lines of evidence have confirmed that inflammation could be induced by the burden of ROS and doxorubicin. Doxorubicin-dependent cell death induces the release of high-mobility group protein B1 (HMGB1) which targets Toll-like receptors 2 and 4 (TLR2 and TLR4). These membrane receptors, in turn, promote immune responses by upregulating the transcription factor NF-*κ*B and then upregulate the expressions of inflammatory factors [29]. Our study also showed infiltration of inflammatory cell in cardiac and hepatic tissue after doxorubicin treatment in HE staining, and this could be reduced by hydrogen-rich saline treatment. In addition, we detected TNF-a, IL-1*β*, and IL-6 levels in cardiac and hepatic tissue; meanwhile, we found that hydrogen-rich saline significantly decreased the expressions of those cytokines, suggesting that hydrogen-rich saline may reduce heart and liver injury by reducing inflammatory responses.

During the past several decades, lots of studies have indicated that DOX-induced cytotoxicity is associated with cell apoptosis from extrinsic and intrinsic signaling pathways. In the extrinsic pathway, the binding of death ligands (FasL, TNF*α*, and TRAIL) with their receptors induces recruitment and activation of caspase-8, which subsequently activates downstream effector caspases such as caspase-3. The intrinsic pathway is regulated by the members of the Bcl-2 family, enhancing apoptosis via inhibition of antiapoptotic Bcl-2 proteins or activation of proapoptotic Bax and Bak. [30]. We investigated the effects of hydrogen-rich saline on doxorubicin-induced cell apoptosis. The TUNEL finding showed that hydrogen-rich saline treatment significantly ameliorated cell apoptosis in both cardiac and hepatic tissues. In addition, we detected the expressions of the Bax/Bcl-2, cleaved C8, and cleaved C3 in three groups, and the western blotting results showed that they were all decreased with the hydrogen-rich saline treatment. These findings may support the amelioration of heart and liver function and mortality by hydrogen-rich treatment.

In conclusion, our present study investigates the potential mechanism of the protective effect of hydrogen-rich saline on doxorubicin and demonstrates that hydrogen-rich saline treatment could inhibit the inflammatory TNF-*α*/IL-6 pathway, increase the cleaved C8 expression and Bcl-2/Bax ratio, and attenuate cell apoptosis in both heart and liver tissue. Due to its safety, efficacy, and convenience, intraperitoneal injection of hydrogen-rich saline should be considered as a potential therapy for heart and liver injury caused by doxorubicin.
