# Corrigendum to “Hydrogen-Rich Saline Attenuates Cardiac and Hepatic Injury in Doxorubicin Rat Model by Inhibiting Inflammation and Apoptosis”

**DOI:** 10.1155/2017/3675910

**Published:** 2017-10-17

**Authors:** Yunan Gao, Hongxiao Yang, Yanbin Fan, Lin Li, Jiahui Fang, Wei Yang

**Affiliations:** ^1^Department of Cardiology, The Fourth Affiliated Hospital of Harbin Medical University, 37 Yiyuan Street, Harbin, Heilongjiang 150001, China; ^2^Department of Cardiology, The First Affiliated Hospital of Harbin Medical University, 23 Youzheng Street, Harbin, Heilongjiang 150001, China

In the article titled “Hydrogen-Rich Saline Attenuates Cardiac and Hepatic Injury in Doxorubicin Rat Model by Inhibiting Inflammation and Apoptosis” [1], there were errors in the units on the *y*-axes of Figures 1(b) and 2(b), which are corrected as follows.

## Figures and Tables

**Figure 1:**
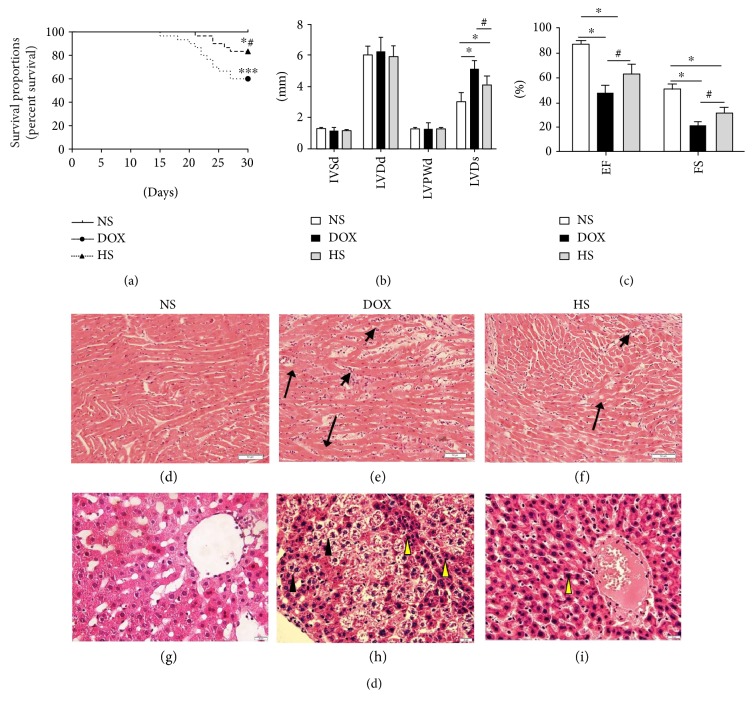
Effects of hydrogen-rich saline treatment on mortality, cardiac dysfunction, and pathological changes. Kaplan-Meier analyses of cumulative survival at 30 days after different treatments (a). The IVSd, LVDd, LVPWd, LVDs, EF, and FS of each rat were assessed (b, c). Morphologic changes of the heart (200x magnification (d, e, f)) and liver (400x magnification (g, h, i)) were processed for HE staining at 30 days (short arrows for infiltrated inflammatory cells and long arrows for focal myolysis; yellow arrowheads for karyopyknosis and black arrowheads for vacuolar degeneration). ^∗^*P* < 0.05 versus NS group; ^∗∗∗^*P* < 0.001 versus NS group; ^#^*P* < 0.05 versus DOX group.

**Figure 2:**
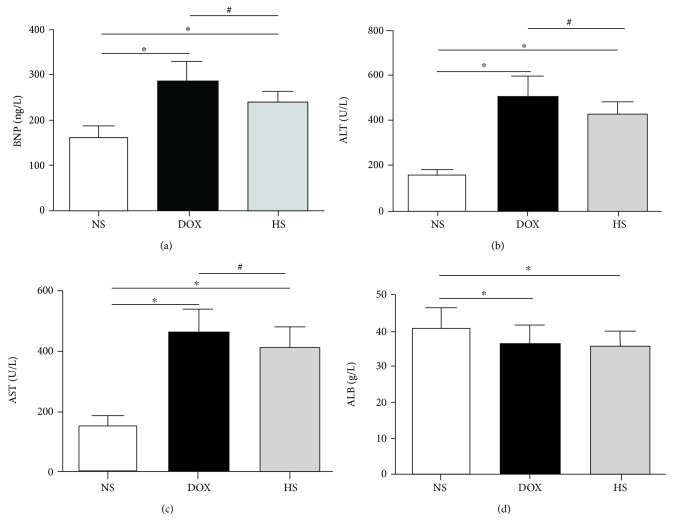
Effect of hydrogen-rich saline on serum parameters. Serum BNP (a), ALT (b), AST (c), and ALB (d) levels in three groups were detected. Data are shown as mean ± SD. ^∗^*P* < 0.05 versus NS group; ^#^*P* < 0.05 versus DOX group.
